# Iron-Catalyzed Synthesis of 4‑oxo-1,3-dioxolanes (DOXs) Using Lactic Acid: From Homogeneous to Heterogeneous Behaviors

**DOI:** 10.1021/acssuschemeng.5c06447

**Published:** 2025-08-26

**Authors:** Massimo Melchiorre, Maria E. Cucciolito, Roberto Esposito, Vincenzo Langellotti, Immacolata Manco, Gregor Schnakenburg, Oreste Tarallo, Federica Tinto, Francesco Ruffo

**Affiliations:** † Dipartimento di Scienze Chimiche, 9307 Università di Napoli Federico II, Via Cintia 21, Napoli 80126, Italy; ‡ ISusChem Srl, Piazza Carità 32, Napoli 80134, Italy; § Consorzio Interuniversitario di Reattività Chimica e Catalisi, Via Celso Ulpiani 27, Bari 70126 Italy; ∥ Institut für Anorganische Chemie, 9374 Rheinische Friedrich-Wilhelms-Universität Bonn, Gerhard-Domagk-Str. 1, Bonn 53121, Germany

**Keywords:** iron(III), α-hydroxy acids, dioxolanes, ketalization, catalysis, catalyst recycling

## Abstract

This work investigates the use of simple iron­(III) and iron­(II) salts (FeSO_4_, Fe­(NO_3_)_3_, Fe­(OAc)_2_, FeCl_2_, FeBr_2_, Fe­(ClO_4_)_2_, Fe­(BF_4_)_2_, FeCl_3_, and Fe­(ClO_4_)_3_) as catalysts for the ketalization of α-hydroxy acids (i.e., lactic and glycolic acids) to obtain 4-oxo-dioxolanes. These compounds find application in polymer science and, more recently, have been proposed as green polar aprotic solvents. Lactic acid and acetone were chosen as benchmark reactants. The results show that catalytic activity depends on the oxidation state of the metal ion, the nature of its counterion, the cosolvent used, and the molar ratio between the reactants. The most effective catalyst was revealed to be iron­(III) perchlorate, which precipitates as iron­(III) lactate that can be recycled in consecutive runs once separated from the reaction mixture. This beneficial prerogative couples the efficiency of homogeneous catalysis with the advantage of heterogeneous catalysis. Compared to other methods proposed in the literature, the proposed process is more sustainable, given its high efficiency, low energy consumption, ease of purification, and the absence of auxiliary substances and byproducts.

## Graphical abstract

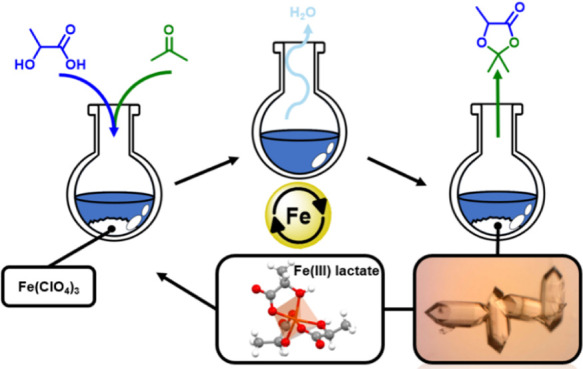

## Introduction

Lactic acid (LA) is a well-established bulk chemical that is mainly produced by industrial carbohydrate fermentation (e.g., glucose, sucrose, galactose).^1^ It is a versatile chemical platform and has several applications in the food industry, detergents, cosmetics, and pharmaceutics.^2^ In 2022, the market volume of LA amounted to approximately 1.5 million tons worldwide.^3^ In the near future (2030), its global production trend is expected to increase to up to 2.8 million metric tons, driven also by its use as a monomer precursor for the synthesis of poly­(lactic acid) (PLA).^4^ PLA is a compostable polymer, and its use for packaging and manufacturing goods has strongly increased, thanks also to the European directive on single-use plastics (Directive 2019/904), which aims at the replacement of petroleum-based polymers.^5^

Although PLA can be produced by its direct polycondensation (*i*, Scheme 1), the stoichiometric production of water determines the instauration of chemical equilibria, ending up with a lower control on polymer properties (e.g., dispersity index, molecular weights). To overcome this aspect, PLA is typically obtained by ring-opening polymerization (ROP)^6^ of lactides (*ii*, Scheme 1), promoted by metal catalysts, typically based on tin­(II) and zinc­(II), but also early transition metals, like Zr­(IV), proved to be suitable for this reaction. An alternative route to PLA was explored using 1,3-dioxolan-4-ones (DOLOs or DOXs) as monomers for homo- and copolymerization reactions promoted by Al­(III) salen catalysts (*iii*, Scheme 1).^7–9^

**Scheme 1:**
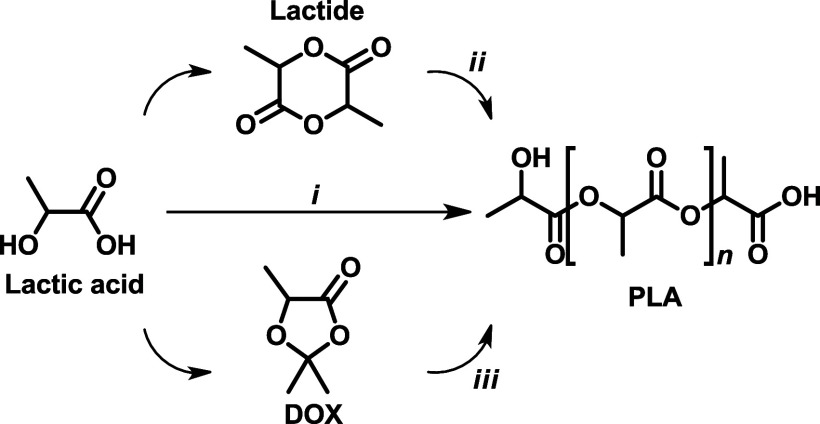
PLA Polymerization Approaches

The use of DOXs as monomers is valuable, especially if typical monomers for ROP (α-hydroxyacid cyclic dimers) are hardly available, as in the case of *rac*- and *meso*-mandelides (e.g., 3 days under reflux in xylene with an acidic catalyst).^10^ Despite their ability to homopolymerize under specific conditions, DOXs are considered as nonhomopolymerizable monomers by cationic ring-opening polymerization (CROP) and are widely investigated to achieve co- and terpolymers with tunable properties (Scheme 2).^11–14^ For instance, the presence of the acetal motifs can improve biocompatibility, as, depending on their composition, the obtained copolymers can be susceptible to hydrolysis even in natural conditions like marine environments.^15^

**Scheme 2:**
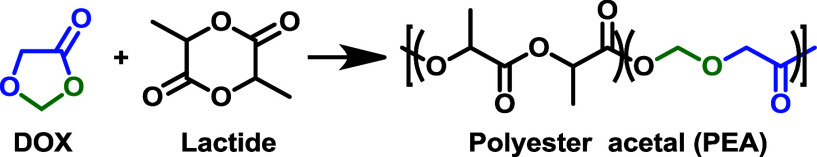
DOXs Copolymer Example

Besides their applications in polymer science, DOXs derived from α-hydroxy acids recently proved to be suitable reaction media for catalytic and stoichiometric reactions,^16^ electrolyte formulations for energy storage devices,^17,18^ as well as solvents for cultural heritage restoration,^19^ paving the way for their investigation as drop-in replacements for hazardous, fossil-based, and persistent dipolar aprotic solvents.^20,21^

However, although this class of compounds has been known for more than a hundred years, and despite their relatively wide exploitation in polymer science and more recently as biobased solvents, to the best of our knowledge, there is no focus in the literature on the improvement of their synthesis. Most of the reported procedures still rely on the use of strong Brønsted acids (e.g., *p*-toluenesulfonic acid, sulfuric acid) with high loading (up to 10%_mol_) and the use of hazardous solvents (e.g., benzene) for prolonged reaction times.^8,9,22–25^

Since DOXs are ketals of α-hydroxy acids with mixed lactone-ether functionality, and given the high performance of iron­(III) catalysts to promote the glycerol ketalization,^26–28^ we decided to explore the use of iron-based Lewis acid catalysts for the synthesis of 2,2,5-trimethyl-1,3-dioxolan-4-one (LA-Me,Me), starting from lactic acid and acetone in nonequilibrium conditions (Scheme 3).

**Scheme 3:**
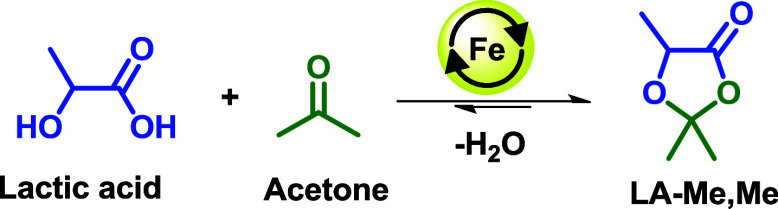
Benchmark Catalysis: Lactic Acid Ketalization with Acetone

Here, we present a comprehensive investigation focused on iron catalysts with different oxidation states and various counterions, alongside the optimization of different reaction parameters, which includes screening of several cosolvents and molar ratios. Among the tested catalysts, Fe­(III) perchlorate proved to be the most performing under optimized conditions, and it was possible to recover an iron-containing spent catalyst that remained active over three catalytic runs. Upon thorough characterization and crystallization, this pale-yellow compound was found to be Fe­(III) lactate, whose structure has never been reported in the literature so far.

The proposed method represents a convenient strategy for the synthesis of DOXs. It exploits Fe­(III) as an abundant and eco-friendly Lewis acid catalyst,^29–31^ involving an easy separation step, and gives access to its immediate recycling.

## Results and Discussion

### Catalysts Screening and Reaction Conditions Optimization

Ketalization is an equilibrium reaction that involves the production of a water molecule. To shift the equilibrium toward the formation of the product, the Dean–Stark apparatus (D–S) was used. Moreover, the ketalization of α-hydroxy acids (AHAs) is complicated by the presence of a parasite self-esterification reaction, which is also promoted by acid catalysts and favored by water removal. A first screening of iron­(II) and iron­(III) salts was performed to evaluate the effects of the oxidation state and of the counterion. Lactic acid conversion, LA-Me,Me yield, and ketalization selectivity are reported in Figure 1.

**Figure 1:**
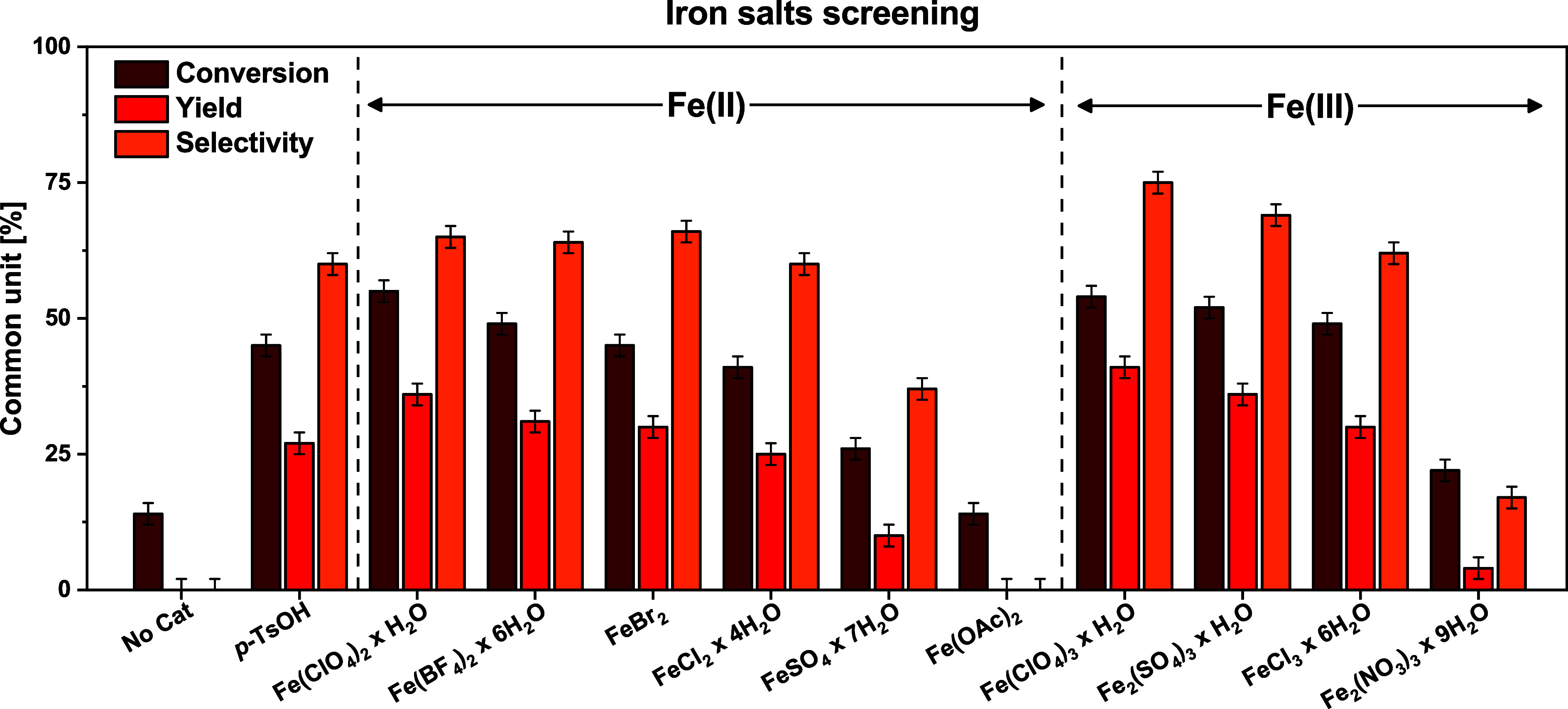
Reaction conditions: D–S apparatus, reflux 4 h, cat 0.05%_mol_, molar ratio 1:4, cosolvent Petr. Eth. 40–60 1:1 in volume vs acetone.

Both oxidation states appeared to be active. Comparing Fe­(II) and Fe­(III) salts with the same counterions (e.g., SO_4_^2–^, Cl^–^, ClO_4_^–^), better results were achieved with Fe­(III). This is reasonably due to the higher Lewis acidity of the Fe­(III) ion compared to the Fe­(II) ion, which improves its catalytic activity. Moreover, the role of the counterion, which is a key aspect in Lewis acid catalysis,^32,33^ is highlighted for both Fe­(II) and Fe­(III) ions. Comparing the trends going from the less basic anion to the more basic (ClO_4_^–^ ∼ BF_4_^–^ < Br^–^ < Cl^–^ < SO_4_^2–^ < OAc^–^), the yield is decreasing for both Fe­(II) and Fe­(III), suggesting a negative correlation between the basicity of the anion and the activity of the catalysts. However, the low activity in the presence of the very few basic NO_3_^–^ ion (basicity Cl^–^ < NO_3_^–^ < SO_4_^2–^) seems not to fit this trend. This can suggest a double effect on the decreasing activity ability of the counterion, being a combination of its Brønsted basicity and its chelating/coordinating ability. Eventually, in the presence of hydrated Fe­(ClO_4_)_3_, the reaction proceeded in higher yield and selectivity compared to those obtained with *p*-TsOH. Therefore, Fe­(III) perchlorate was chosen for the subsequent optimization.

Due to the use of the Dean–Stark apparatus (D–S) to remove water from the reaction mixture, a screening of different cosolvents was performed. Indeed, the use of a hydrophobic blend of solvents is crucial for using the D–S apparatus and shifting the equilibrium toward the product formation. This condition has also been experimentally proven using acetone without any cosolvent (Figure 2, “None” bars), achieving only 17% of yield.

**Figure 2:**
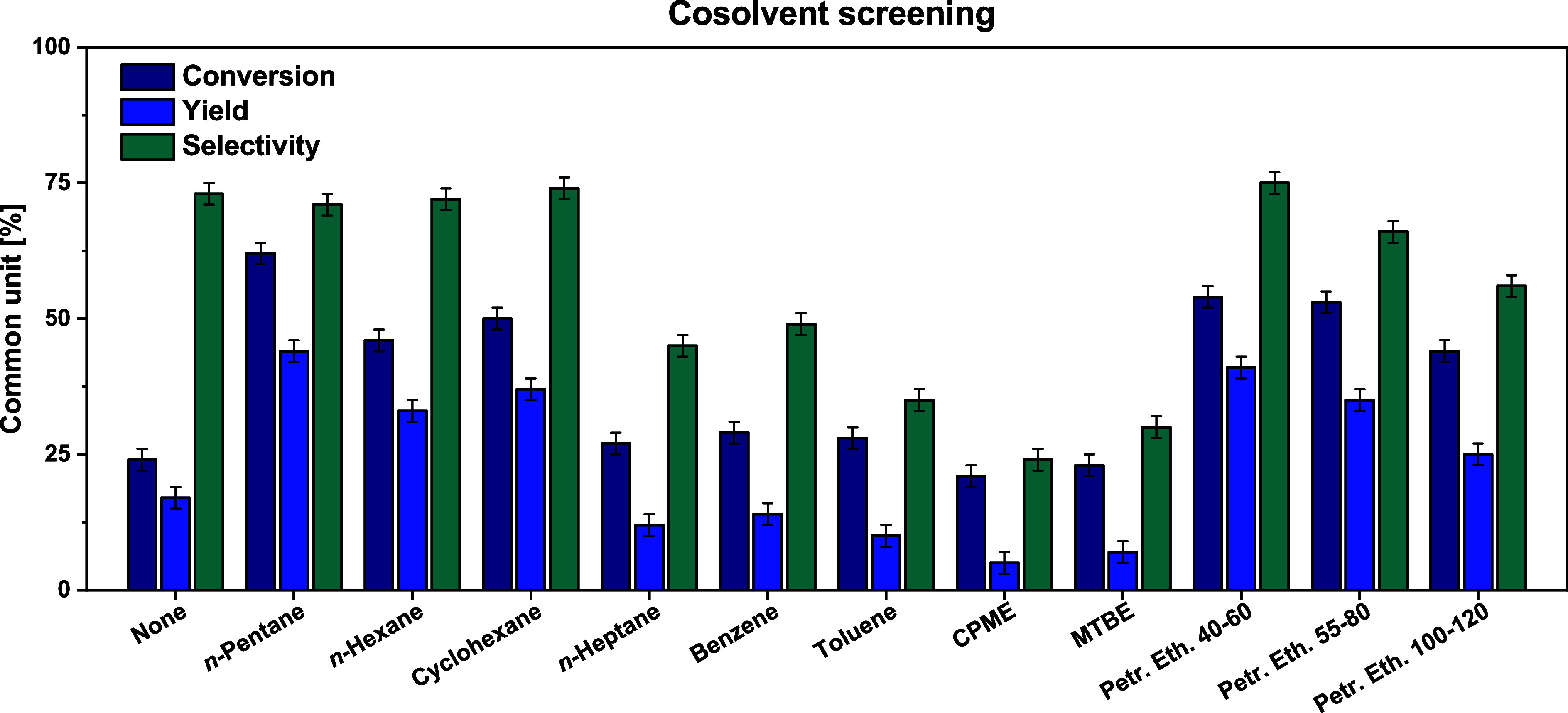
Reaction conditions: D–S apparatus, reflux 4 h, Fe­(III) perchlorate 0.05%_mol_, molar ratio 1:4, cosolvent 1:1 in volume vs acetone.

As shown in Figure 2, ethers or aromatic solvents tend to worsen the overall results. This could be due to specific catalyst-solvent interaction in the case of ethers, and due to the higher mutual miscibility of water in aromatics.^34^ Among the aliphatic solvents, the boiling point seems also to play a relevant role. Among different petroleum ethers boiling in the range of 40–120 °C, the best result was achieved with the more volatile solvent (40–60 °C). The same trend was observed with *n*-pentane (bp 36 °C), *n*-hexane (bp 68–69 °C), and cyclohexane (bp 80 °C), which displayed better performance compared to *n*-heptane (bp 98–99 °C).

This trend could appear counterintuitive, since higher boiling temperatures are expected to remove water from the reaction mixture more efficiently. However, higher temperatures could improve the mutual solubility of water in the reaction mixture,^35^ and, in addition, the standard formation enthalpy of ketals is typically reported as exothermic.^36^

Defined the best catalyst and cosolvent, a screening of the reagents’ molar ratio (MR) was performed. To avoid effects related to the change of catalyst and lactic acid concentration and to mitigate changes of composition during the reaction time, the total volume of the reaction mixture was kept constant (24 mL). The molar ratio was adjusted using acetone/petroleum ether mixtures with a proper volume ratio, and the D–S tube was filled with the same acetone/petr. ether mixture (details in the Experimental Section). The results are reported in Table 1.

**Table 1:** Lactic Acid: Acetone Molar Ratio (MR) Screening^a^

**Entry**	**Molar ratio [MR]**	**Ace:Cosolv [mL]**	**Conversion [%]**	**Yield [%]**	**Selectivity [%]**
1	1:4	12:12	54	41	75
2	1:2	18:6	83	65	79
3	1:1	21:3	76	63	83

a Reaction conditions: D–S apparatus, reflux 4 h, Fe­(III) perchlorate 0.05%_mol_, Petr. Eth. 40–60 to acetone volume ratio changes according to the desired molar ratio (see Experimental Section). Evaluated by ^1^H NMR, error ± 2%.

Within this screening, the condition of Entry 3 was the most performing, thus also maximizing the atom economy of the reaction. The biphasic reaction mixture at the end of the reaction was treated with a few drops of acetone to homogenize the liquid system, and at the same time, a pale-yellowish solid precipitated (Figure S1). Eventually, the reaction conditions in Entry 3 were used to isolate the product by vacuum distillation, achieving a 60% yield in good agreement with NMR data. Overall, the defined *optimized conditions* consist of a stoichiometric molar ratio (MR 1), an aliphatic solvent (petr. eth. bp 40–60 °C), and Fe­(ClO_4_)_3_ as catalyst precursors at low loading.

### Reaction Mixture and Solid Precipitate Characterization

To understand the catalyst behavior, the isolated liquid and solid phases were subjected to further investigations. The iron­(III) content in the liquid phase was evaluated using UV–vis spectroscopy with a previously reported method.^37^ From this analysis, the final iron content in the liquid phase was found to be 5 ppm (details in Figures S2 and S3). The isolated solid was subjected to UV–vis (water solution, Figure S4), FT-IR (Nujol method, Figure S5), and wide-angle X-ray diffraction (WA-XRD, Figure S6). Moreover, to verify the presence of organic fragments, part of the solid was treated with a solution of sodium sulfide in D_2_O to precipitate iron sulfide and analyze the resulting solution by ^1^H NMR spectroscopy (Figure S7). All the investigations performed pointed out that the isolated solid was an iron compound containing a lactate fragment as an anion (individuated by ^1^H NMR).

Considering the microcrystallinity of the isolated solid, many recrystallization attempts were performed to obtain a single crystal suitable for X-ray analysis. However, none of the used solvent/nonsolvent mixtures and crystallization strategies provided suitable-sized crystals. Unexpectedly, serendipitous crystallization occurred in an aged reaction mixture (high MR), which initially did not present any precipitate. After some months at ambient temperature, a few small single crystals (0.2–0.5 mm in length) were obtained, and one of them was selected for the single-crystal X-ray analysis (Figure 3a).

**Figure 3:**
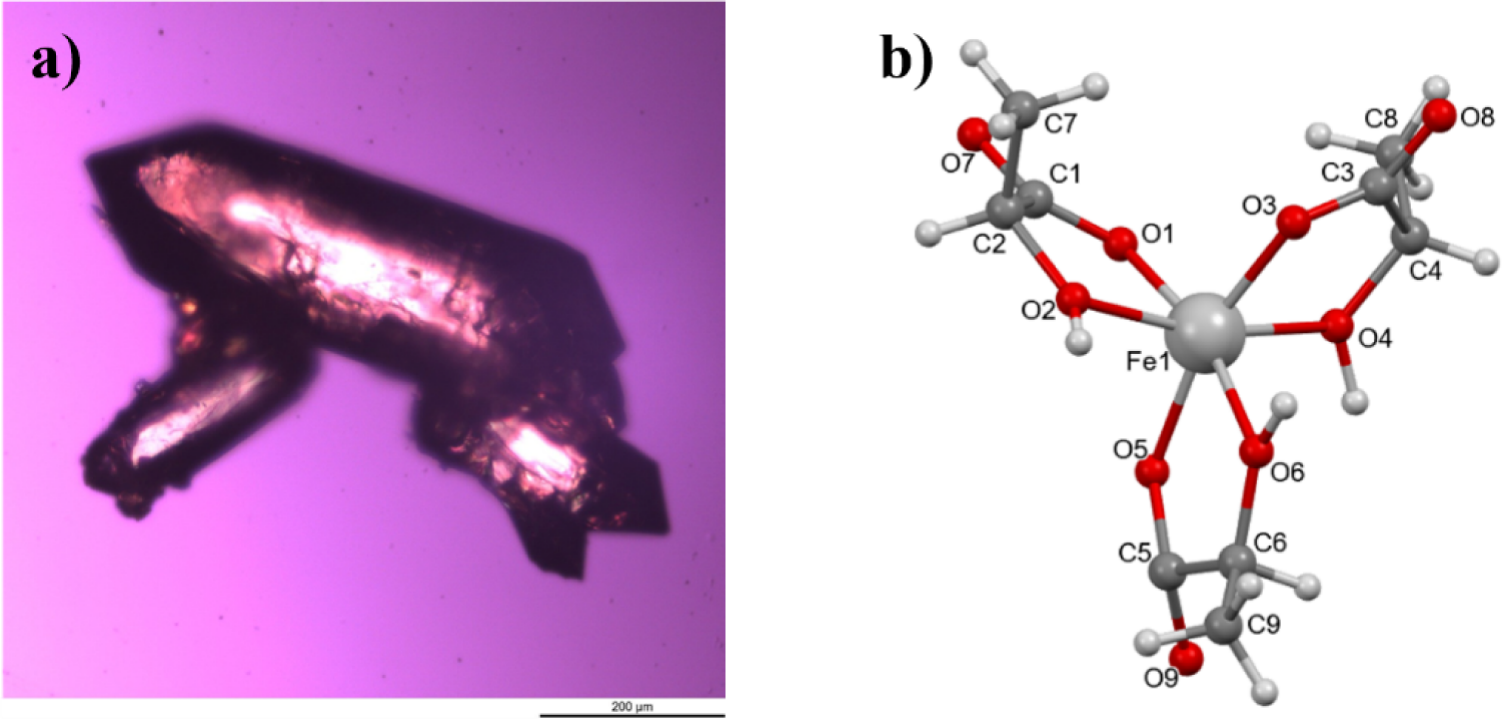
(a) Recovered crystals photo under microscope light. (b) Molecular structure of the recovered solid compound **1-Fe** (Mercury program,^38^ ball-and-stick style).

Metal complexes with lactic acid crystallize with great difficulty, and a limited number of structural X-ray data in the literature were found. Some examples with tris-lactate as chelating ligands have been reported for neutral In­(III),^39^ anionic Ti­(IV)^40^ and Al­(III),^41^ but no previous structure of iron­(III) lactate has been reported in the literature so far.

The structural analysis of the recovered compound **1-Fe** showed that it is neutral and that the Fe­(III) cation is coordinated in a bidentate manner by three lactate anions (Figure 3b). In the coordination sphere, the carboxylic oxygen atoms are deprotonated, while the alcoholic groups of lactic acids are protonated. The crystal packing is stabilized by strong OH···O=C hydrogen bonds, where the alcoholic oxygen acts as a donor toward the carboxyl oxygen of an adjacent molecule.

This unexpected outcome led to two relevant benefits of the catalytic system: (i) the spent catalyst can be easily separated from the reaction mixture, and (ii) the spent catalyst can be reused to evaluate its effective recycling.

### Catalyst Recycling and Substrate Scope

A series of consecutive ketalization reactions of lactic acid with acetone was carried out under previously optimized conditions. After each run, the catalyst was recovered by decantation, washed with acetone, and reused in subsequent runs without any reactivation process. Overall results are collected in Table 2, while relevant ^1^H NMR spectra are reported in Figure S8. The first set of experiments was performed starting with 0.05%_mol_ of catalyst, and a sharp decay of yield was obtained after Run 0. To mitigate the effect of any mass loss related to the compound manipulations, a further set of experiments was performed, increasing the catalyst loading up to 0.50%_mol_. Within this test, the overall conversions exhibited some fluctuation, but the selectivity showed an increasing trend, achieving 95% in the second run (Entries 1–3 of Table 2). This behavior could be due to the transformation of the catalyst precursor from iron­(III) perchlorate to iron­(III) lactate. This transition likely induces a shift in selectivity, driven by the counterion effect, that plays a key role in the catalytic pathway. Another explanation could be the *in situ* formation of perchloric acid during Run 0, which is responsible for Brønsted acid-mediated processes. In subsequent runs, the suppression of this contribution, due to the use of a different catalyst precursor, excludes any Brønsted acid-promoted process.

**Table 2:** Catalyst Recycling Test^a^

**Entry**	**Run #**	**Cat loading [%**_**mol**_]	**Conversion [%]**	**Yield [%]**	**Selectivity [%]**
1	Run 0	0.50	84	62	73
2	Run 1	-	71	55	77
3	Run 2	-	79	75	95

a Reaction conditions: D–S apparatus, reflux 4 h, cat Fe­(III) perchlorate hydrate, MR 1 (Petr. Eth. bp 40–60 °C: acetone 3:1 in volume). Evaluated by ^1^H NMR, error ± 2%. The isolated, spent catalyst was directly reused for the next run.

Despite that the fate of iron­(III) was assessed without ambiguity, the destiny of perchlorate counterions is still unclear and needs to be defined, although it is clear from FT-IR of the recovered catalyst that it gradually disappears through the runs (Figure S9). To assess the chemical behavior of the catalytic precursor, the free iron­(III) concentration in the crude mixture was followed at different reaction times for Run 0 and after Run 2. The overall results are presented in Figure 4. The iron­(III) concentration showed a diminishing trend, starting from an initial loading of ≈1900 ppm and reaching 60 ppm within 4 h, and even lower after Run 2 (18 ppm). For this set of experiments, a higher catalyst loading was used (0.5%_mol_) and all the volatile phases were evaporated before the spectrophotometric titration.

**Figure 4:**
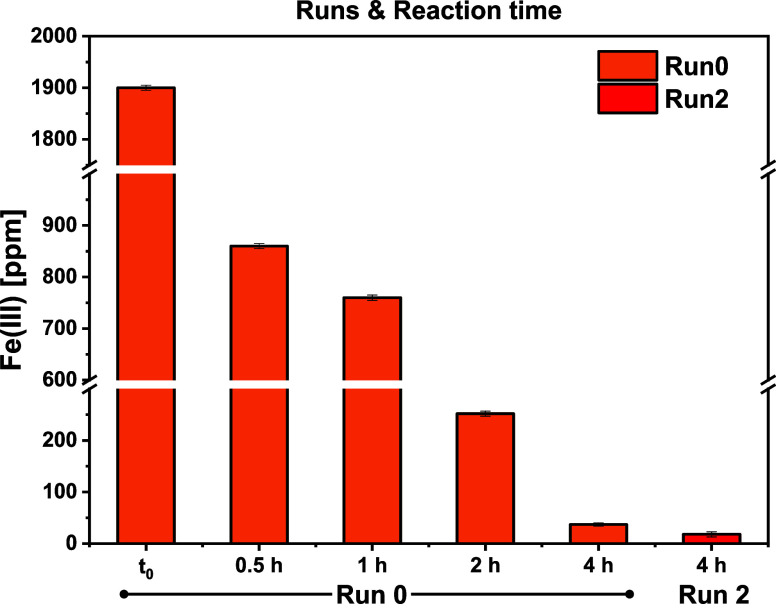
Fe­(III) concentration at different reaction times and different runs.

This investigation confirmed the gradual conversion of iron­(III) perchlorate during the reaction progress and the inherent formation of iron­(III) lactate, insoluble in the final crude mixture. To evaluate the robustness of the proposed method, the substrate scope was extended to other carbonyl compounds and glycolic acid as an alternative α-hydroxy acid. The results are reported in Table 3.

**Table 3:** Substrate Scope^a^

**Entry**	**AHAs**	**Carbonyl compound**	**Cat loading** [mol %]	**Conversion [%]**	**Yield [%]**	**Selectivity [%]**
1	LA	Cyclopentanone	0.05	62	47	81
2	LA	Benzaldehyde	0.05	57	44^b^	78
3	LA	Trioxane	0.50	99	81	82
4	GA	Acetone	0.05	63	63	>99

a Reaction conditions: D–S apparatus, reflux 4 h, cat Fe­(III) perchlorate hydrate, MR 1, cosolvent petr. eth. bp 40–60 °C. Results evaluated by ^1^H NMR, error ± 2%. LA: lactic acid, GA: glycolic acid.

b Diastereomers ratio 1:2.

Overall, the method was easily adapted for high-boiling-point ketones (Entry 1, ^1^H NMR spectrum in Figure S10), aromatic aldehydes (Entry 2, ^1^H NMR spectrum in Figure S11), and formaldehyde cyclic trimer (Entry 3, spectrum in Figure S12). Among other AHAs, the method was suitable and highly selective with glycolic acid (Entry 4, spectrum in Figure S13), while in combination with more sterically hindered substrates (i.e., mandelic acid, α-hydroxyisobutyric acid—spectra in Figures S14 and S15), the catalytic system was not effective (yield <5%).

### Proposed Catalytic Pathway

Robust computational and experimental investigations should be conducted to properly evaluate the catalytic cycle for this reaction. However, some speculations can be made based on previous results.^27^

Considering the formation of compound **1-Fe** as shown by the X-ray structure, it is possible to consider some equilibria within the first run that lead to its formation. Despite perchloric acid being a stronger acid than lactic acid, the equilibrium depicted in Scheme 4 could be pushed toward the formation of **1-Fe** due to its precipitation. As a matter of fact, similar conditions are used in the traditional production of perchloric acid: alkali perchlorates (e.g., NaClO_4_) are treated with hydrochloric acid and solid alkali chloride precipitate (e.g., NaCl).^42^

**Scheme 4:**
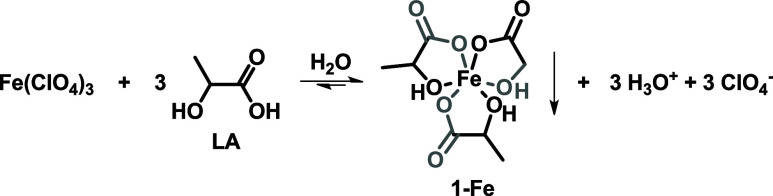
Proposed Equilibrium for the Formation of Compound **1-Fe**

As mentioned before, the catalytic activity in the first run could also be attributed to a Brønsted contribution of perchloric acid. Residual water traces in the reaction mixture, despite the D–S apparatus, may facilitate this process by allowing the formation of hydronium perchlorate. In addition, continuous boiling with the D–S apparatus could also strip perchloric acid and water, as they form an azeotrope of $70{\%}_{{HClO}_{4}}-30{\%}_{H_{2}O}$ .^42^

Anyway, starting from the second run, the contribution of any perchloric acid is excluded, and a possible catalytic cycle starting from **1-Fe** is proposed (Scheme 5).

**Scheme 5:**
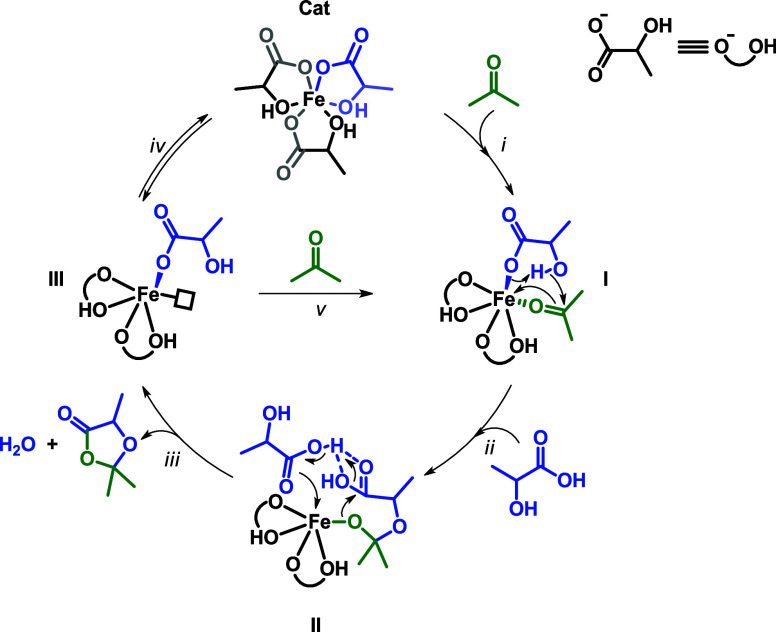
Proposed Catalytic Pathway: (*i*) Interchange Hydroxyl Group and Acetone; (*ii*) Concerted Intramolecular Nucleophilic Attack and Proton Transfer; (*iii*) Lactic Acid Assisted Cyclization and Water Elimination; (*iv*) Hydroxyl Group Coordination Equilibria; (*v*) Acetone Coordination

The initial step involves an exchange between the hydroxyl group moiety of a lactate ligand and acetone. This step occurs with an interchange-like (*i*) or dissociative (*iv, v*) mechanism involving fluxional equilibria of lactate-neutral moieties (OH). Once activated by the iron center, the electrophilic carbon of acetone is subjected to nucleophilic attack from the free −OH moiety of the open-lactate ligand (in blue) and concerted proton transfer. Compared to an intermolecular attack from bulk lactic acid, the intramolecular attack could be favored because of the proximity of the carboxylate moiety of the same lactate, which can act as a base. Indeed, in the case of an intermolecular nucleophilic attack, the free hydroxyl group in the proximity of acetone would lead to a crowded inner sphere (example in Scheme 6).

**Scheme 6:**
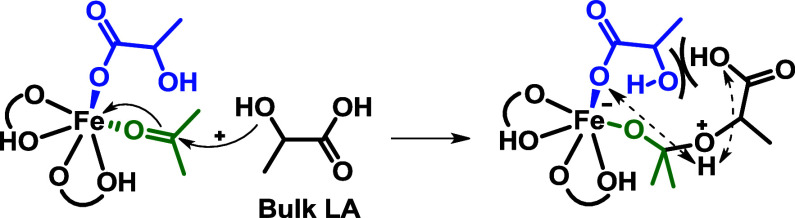
Example of Intermolecular Nucleophilic Attack from Bulk LA

The subsequent proposed step is assisted by a bulk LA, which acts as a proton source to activate the carboxylic carbon for its nucleophilic attack, leading to the concerted product formation, the elimination of water, and the coordination of a new lactate ligand to the iron ion that regenerates the catalyst (*iii*).

According to previous results and the proposed catalytic pathway, the presence of coordinating solvents like ethers (e.g., MTBE, CPME) might compete with the acetone in the coordination to the metal center, while a stronger acidic Fe­(III) metal center might be responsible for larger polarization of the coordinated acetone, leading to a more pronounced electrophilic character of its carbonylic carbon.

## Experimental Section

### General

All solvents and reagents were purchased from Merck Life Science Srl (Milano, Italy) and used without further purification. NMR spectra were recorded using a Bruker 400 or Varian 500 MHz spectrometer with deuterated solvent (mainly CDCl_3_ and D_2_O). Most of the iron salts have been used in their hydrated form (FeCl_2_·4H_2_O, FeCl_3_·6H_2_O, Fe­(ClO_4_)_2_·*x*H_2_O, Fe­(ClO_4_)_3_·*x*H_2_O, Fe­(NO_3_)_3_·9H_2_O, Fe­(SO_4_)·7H_2_O, Fe_2_(SO_4_)_3_·*x*H_2_O) since the reactions have been performed in an open-air environment, the solvents have not dried before use, and the used lactic acid contains a certain amount of water (≈10%_wt_). Only Fe­(OAc)_2_ and FeBr_2_ have been employed in their anhydrous forms, as these are the most commercially accessible variants. For an undefined hydrate formula (*x*H_2_O), molecular weights on an anhydrous basis have been used. **Warning**: perchlorate iron salts are strong oxidizers that pose fire and explosion hazards and must be handled with extreme caution under controlled conditions.

UV–vis spectra were recorded by using a JASCO V-530 UV/vis spectrophotometer. FT-IR spectra were recorded on a Nicolet Avatar 360 FT-IR spectrometer in the range of 4000–400 cm^–1^, with a 2 cm^–1^ resolution. Samples were prepared by using the Nujol method and KBr plates. Wide-angle X-ray diffraction (XRD) patterns of the samples were recorded by using a Philips PW1830 automatic powder diffractometer. The instrument operated in the θ/2θ Bragg–Brentano geometry, utilizing nickel-filtered Cu Kα radiation. A continuous scan was performed over a 2θ range of 1.6–50°. The samples were mounted in specimen holders with a thickness of 2 mm for the analysis.

### Benchmark Ketalization Reaction

To avoid both different catalyst concentrations in the molar ratio screening and the variable efficiency of the water removal system due to different total volumes, it was decided to use a fixed volume of a cosolvent–acetone mixture (24 mL) for a defined amount of lactic acid. Therefore, an appropriate cosolvent–acetone mixture was prepared (≈100 mL) with a proper volume ratio according to the desired molar ratio (MR—lactic acid:acetone) to be investigated (e.g., MR 1:4 uses a 1:1 V_cosolv_/V_ace_ mixture, MR 1:2 uses a 3:1 V_cosolv_/V_ace_ mixture, MR 1:1 uses a 7:1 V_cosolv_/V_ace_). In a 50 mL round-bottom flask equipped with a Dean–Stark apparatus and a condenser, 4.0 g (40 mmol) of lactic acid 90%_wt_, and 0.05%_mol_ of the chosen catalyst precursor (e.g., Fe­(ClO_4_)_3_·*x*H_2_O 0.02 mmol, 7 mg) were combined with 24 mL of the cosolvent–acetone mixture (1:1 v/v, 0.16 mol). The mixture was refluxed under vigorous stirring, and the reaction proceeding was highlighted by the formation of water into the Dean–Stark trap, previously filled with the same cosolvent–acetone mixture used for the reaction. After the chosen reaction time (e.g., 4 h), a sample was collected from the crude reaction and analyzed by ^1^H NMR. An explanatory ^1^H NMR spectrum is reported in Scheme 7. Conversion, yield, and selectivity have been evaluated with the following equations (Equations 1–3). 2,5,5-trimethyl-1,3-dioxolan-4-one product signals: ^1^H NMR (400 MHz, CDCl_3_) δ 4.48 (q, *J* = 6.7 Hz, 1H), 1.61 (s, 3H), 1.54 (s, 3H), 1.48 (d, *J* = 6.7 Hz, 3H).

1 $$\mathrm{Conversion}\;(\%)=\frac{\int a^{\prime }+\int a^{\prime \prime }}{\int a+\int a^{\prime }+\int a^{\prime \prime }}\cdot 100$$

2 $$\mathrm{Yield}\;(\%)=\frac{\int a^{\prime }}{\int a+\int a^{\prime }+\int a^{\prime \prime }}\cdot 100$$

3 $$\mathrm{Selectivity}\;(\%)=\frac{\mathrm{Yield}}{\mathrm{Conversion}}\cdot 100$$

**Scheme 7:**
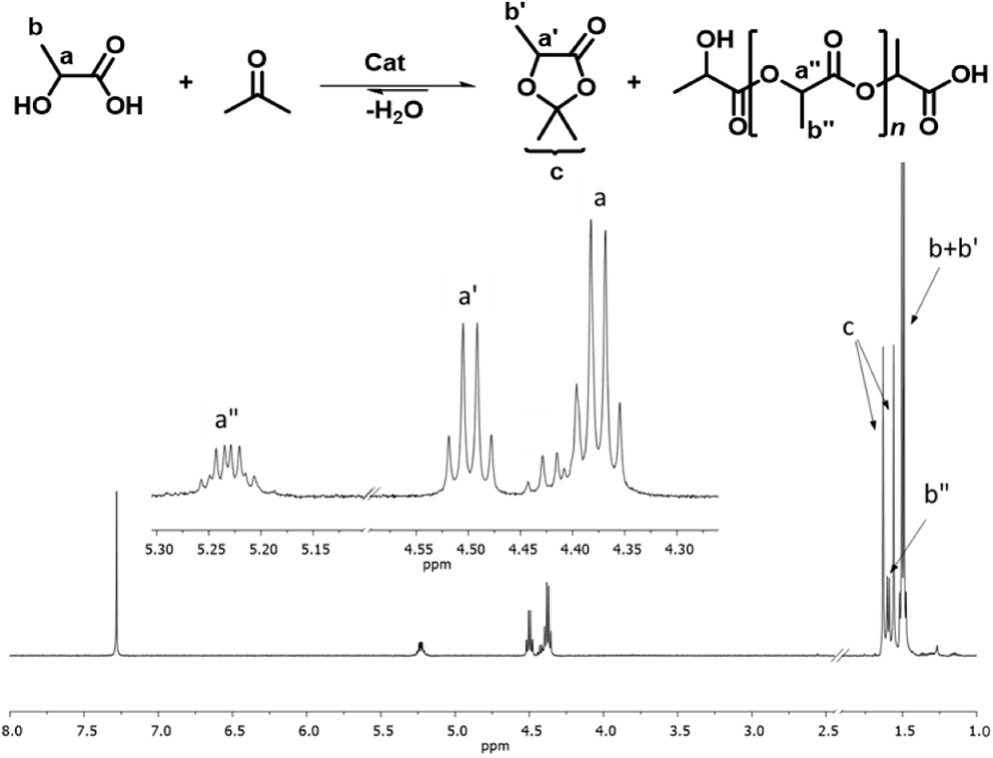
^1^H NMR in CDCl_3_ of the Crude Mixture

### Iron­(III) Content Measurement

The free iron­(III) content in the samples was determined using UV–Vis spectroscopy by forming and titrating the iron thiocyanate complex [Fe­(SCN)_6_]^3–^. Quantification was achieved via a standard addition method. After the catalytic cycle, the solid residue was removed by centrifugation, and the supernatant was filtered through a 0.22 μm PTFE syringe filter. From the obtained solution, 1.00 mL was withdrawn and diluted to 2.00 mL using a solution containing 0.20 M potassium thiocyanate in a 1:1 acetone/water mixture, with the water component buffered at pH 2.5. This mixture was labeled *Sample A*. A *Blank* was prepared by diluting 1.00 mL of the filtered reaction solution to 2.00 mL with the same acetone/water buffer solution at pH 2.5. The UV–Vis spectrum of *Sample A* was recorded against the blank, and the absorbance at 484 nm was measured. Subsequently, 5 μL of a 14.4 mM Fe­(III) solution was added to *Sample A*, and the UV–Vis spectrum was again recorded against the blank. The absorbance at 438 nm was registered, and this process was repeated twice more to create a four-point calibration curve. This calibration was linearly fitted with an *R* ^2^ value greater than 0.995. The initial iron­(III) concentration was calculated using linear regression. Examples of absorbance spectra and calibration lines are reported in the Supporting Information.

### Single Crystal X-ray Analysis

A clear, yellowish colorless block of **1-Fe** (0.24 × 0.22 × 0.16 mm) was mounted on a Bruker D8 Venture 4-circle Kappa diffractometer equipped with a low-temperature device (100(2)­K, Oxford Cryostream 800 series, Oxford Cryosystems) by using Mo-*K*α radiation (λ = 0.71073 Å, Helios mirror optics) and a PHOTONIII/C14 CMOS detector system. Intensities were measured by fine-slicing φ- and ω-scans and corrected for background, polarization, and Lorentz effects. A semiempirical absorption correction from equivalent reflections (multiscan type) was performed by using SADABS (SADABS-2016/2, Bruker AXS, **2016**).

Structure solution was done using intrinsic phasing methods included in the SHELXT program system^43^ and refined by full-matrix least-squares/difference Fourier synthesis with ShelXL-2019/3.^44^ All non-hydrogen atoms were refined anisotropically; hydrogen atoms were placed in geometrically calculated positions and included using a riding model on the bound carbon atoms and relative isotropic displacement parameters.

The absolute configuration was verified by a Bijvoet-Pair Analysis via Bayesian Statistics; the P2/P3 probability of correct absolute configuration was determined to be 100%; the P3­(rac-twin) and P3­(false) probabilities were calculated to 0%.^45^ Details of the crystal data and structure refinement parameters are reported in Table S1.

## Conclusions

This study demonstrates that simple iron­(III) salts can be effective catalyst precursors to promote the ketalization of α-hydroxy acids with some carbonyl compounds. An in-depth investigation using lactic acid and acetone as benchmark reactants disclosed that the reaction led to the formation of iron­(III) lactate, which was insoluble in the final reaction mixture but still active in consecutive runs. This beneficial prerogative couples the efficiency of homogeneous catalysis with the advantage of heterogeneous catalysis. Moreover, considering a hypothetical integrated industrial production, the obtained lactate-oligomers are valuable raw materials for lactide production rather than *side-products*. Also, a hydrolytic treatment would give back the starting lactic acid, which might be reused in the ketalization process, achieving total closed-loop recycling of the reaction mixture.

## Supplementary Material



## Notes

Relevant ^1^H NMR, FT-IR, and XRD spectra and other images (PDF)

## Notes

CCDC 2467877 contains the supplementary crystallographic data for this paper, including structure factors. The data can be obtained free of charge from the Cambridge Crystallographic Data Centre via www.ccdc.cam.ac.uk/structures.
