# Supplementary material for: Iron-Catalyzed Synthesis of 4‑oxo-1,3-dioxolanes (DOXs) Using Lactic Acid: From Homogeneous to Heterogeneous Behaviors
Source: ACS Sustain Chem Eng. 2025 Aug 26;13(38):16071–9. doi: 10.1021/acssuschemeng.5c06447 (PMC12486447; doi:10.1021/acssuschemeng.5c06447)
Supplement: Supplementary file 1 [file sc5c06447_si_001.pdf]

Iron-catalyzed synthesis of 4-oxo-1,3-dioxolanes (DOXs) using lactic acid: from homogeneous to heterogeneous behaviors.

Massimo Melchiorre,[a, b] Maria E. Cucciolito,[a, c] Roberto Esposito,[a, c,] Vincenzo Langellotti,[a] Immacolata Manco,[a] Gregor Schnakenburg,[d] Oreste Tarallo,[a] Federica Tinto [a] and Francesco Ruffo [a, c]*

[a] Dipartimento di Scienze Chimiche, Università di Napoli Federico II, Via Cintia 21, 80126, Napoli, Italy.

[b] ISusChem Srl, Piazza Carità 32, 80134, Napoli, Italy.

[c] Consorzio Interuniversitario di Reattività Chimica e Catalisi, Via Celso Ulpiani 27, 70126, Bari, Italy.

[d] Institut für Anorganische Chemie, Rheinische Friedrich-Wilhelms-Universität Bonn, Gerhard-Domagk-Str. 1, 53121 Bonn, Germany.

* roberto.esposito@unina.it

14 Pages; 15 Figures, 2 Tables

Table of contents

Table S1. Details of **1-Fe** crystal data and structure refinement parameters. 3

Figure S1. Photo of crude reaction mixture (**Table 1, Entry 3**) after the addition of acetone.........................4

Figure S2. UV-Vis spectra of crude mixture treated with acidic solution of sodium thiocyanate before (0 ppm) and after the standard addition of Fe(III) chloride solution (2-6 ppm). 4

Figure S3. Data manipulation from **Figure S2**. 5

Figure S4. UV-Vis spectra in water and comparison with $Fe(ClO_4)_3$..5

Figure S5. FT-IR spectra and comparison of Run 0 spent cat (0.5 $\%_{mol}$ cat loading) with the resulting solid obtained by treating iron(III) perchlorate with lactic acid (4 h at reflux in petr. eth. bp 40–60 °C).6

Figure S6. WA-XRD pattern of spent catalyst. 6

Figure S7. ^{1}H NMR spectra in D_2O of the spent catalyst treated with hydrated $Na_2S{\cdot}9H_2O$. 7

Figure S8. Relevant ^{1}H NMR portion of **Entries 1-3** (bottom to top) from **Table 2**. 7

Figure S9. FT-IR analysis after catalytic Run 0 and Run 2 (cat loading 0.5 $\%_{mol}$)..8

Figure S10. ^{1}H NMR spectrum of **Table 3 Entry 1**, ketalization of lactic acid with cyclopentanone. Reaction conditions: D-S apparatus, reflux 4 h, cat Fe(III) perchlorate hydrate, MR 1, cosolvent petr. eth. bp 40–60 °C. **Used signals**: **a''** 5.20 ppm (m, 1H); **a'**, 4.42 ppm (q, J = 6.8 Hz, 1H); **a,** 4.37 ppm (q, J = 6.9 Hz, 1H). Multiple signals at 1.8-1.48 ppm have not been attributed to each compound...9

Figure S11. ^{1}H NMR spectrum of **Table 3 Entry 2**, ketalization of lactic acid with benzaldehyde. Reaction conditions: D-S apparatus, reflux 4 h, cat Fe(III) perchlorate hydrate, MR 1, cosolvent petr. eth. bp 40–60 °C. **Used signals**: **c***, 6.59 and 6.38 ppm (s, 1H – minor and major diastereomer); a'' 5-23 (m, 1H); **a'**, 4.54 (q, J = 6.6 Hz, 1H); **a**, 4.37 (q, J = 6.9 Hz, 1H). 10

Figure S12. ^{1}H NMR spectrum of **Table 3 Entry 3**, ketalization of lactic acid with trioxane. Reaction conditions: D-S apparatus, reflux 4 h, cat Fe(III) perchlorate hydrate, MR 1, cosolvent petr. eth. bp 40–60 °C. Due to high conversion, volatile compounds were gently evaporated before analysis. **Product signals**: ^{1}H NMR (400 MHz, $CDCl_3$) δ 5.53 (s, 1H), 5.41 (s, 1H), 4.29 (q, J = 6.8 Hz, 1H), 1.50 (d, J = 6.8 Hz, 3H). 11

Table S2. Fitting data analysis for the ^{1}H NMRspectrum of **Figure S12**. 11

Figure S13. ^{1}H NMR spectrum of **Table 3 Entry 4**, ketalization of glycolic acid with acetone. Reaction conditions: D-S apparatus, reflux 4 h, cat Fe(III) perchlorate hydrate, MR 1, cosolvent petr. eth. bp 40–60 °C. **Product signals**: ^{1}H NMR (400 MHz, $CDCl_3$) δ 4.34 (s, 2H), 1.59 (s, 6H). 12

Figure S14. ^{1}H NMR spectrum for the ketalization of mandelic acid with acetone. Reaction conditions: D-S apparatus, reflux 4 h, cat Fe(III) perchlorate hydrate, MR 4, cosolvent petr. eth. bp 40–60 °C. **Product signals**: ^{1}H NMR (400 MHz, $CDCl_3$) δ 7.45 (m, 5H), 5.39 (s, 1H), 1.72 (s, 3H), 1.68 (s, 3H). 13

Figure S15. ^{1}H NMR spectrum for the ketalization of α-hydroxyisobutyric acid with acetone.Reaction conditions: D-S apparatus, reflux 4 h, cat Fe(III) perchlorate hydrate, MR 4, cosolvent petr. eth. bp 40–60 °C. **Used signals**: **a** 1.47 (s, 6H – reagent and product); **b,** 1.60 ppm (s, 6H – product). 14

Table S1. Details of **1-Fe** crystal data and structure refinement parameters.

Crystal Habitus	clear yellowish colourless block
Device Type	Bruker D8 Venture
Empirical formula	$C_9H_{15}O_9Fe$
Moiety formula	$C_9H_{15}FeO_9$
Formula weight	323.06
Temperature/K	100.00
Crystal system	trigonal
Space group	$P3_1$
a/Å	8.8693(3)
b/Å	8.8693(3)
c/Å	14.6034(7)
α/°	90
β/°	90
γ/°	120
Volume/Å^3	994.86(8)
Z	3
ρ_{calc}g/cm^3	1.618
μ/mm^{-1}	1.175
F(000)	501.0
Crystal size/mm^3	0.24 × 0.22 × 0.16
Absorption correction	multi-scan
Tmin; Tmax	0.6571; 0.7376
Radiation	MoKα (λ = 0.71073)
2Θ range for data collection/°	5.304 to 55.998°
Completeness to theta	0.958
Index ranges	$-11 \leq h \leq 11$, $-11 \leq k \leq 11$, $-19 \leq l \leq 19$
Reflections collected	23436
Independent reflections	3092 [R_{int} = 0.0723, R_{sigma} = 0.0466]
Data/restraints/parameters	3092/1/179
Goodness-of-fit on F^2	1.193
Final R indexes [I>=2σ (I)]	R_1 = 0.0494, wR_2 = 0.1307
Final R indexes [all data]	R_1 = 0.0499, wR_2 = 0.1309
Largest diff. peak/hole / e Å^{-3}	0.62/-0.51
Flack parameter	0.09(4)
P2(true)	1.000
P3(true)	1.000
P3(rac-twin)	0.000
P3(false)	0.000
CCDC number	2467877

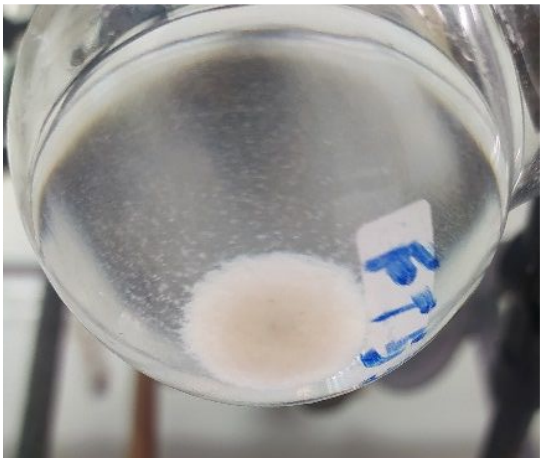

Figure S1. Photo of crude reaction mixture (**Table 1, Entry 3**) after the addition of acetone.

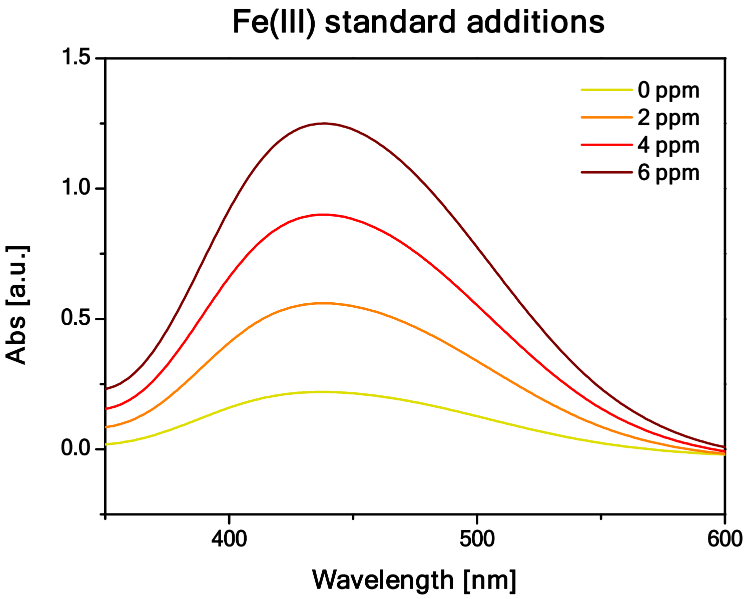

Figure S2. UV-Vis spectra of crude mixture treated with acidic solution of sodium thiocyanate before (0 ppm) and after the standard addition of Fe(III) chloride solution (2-6 ppm).

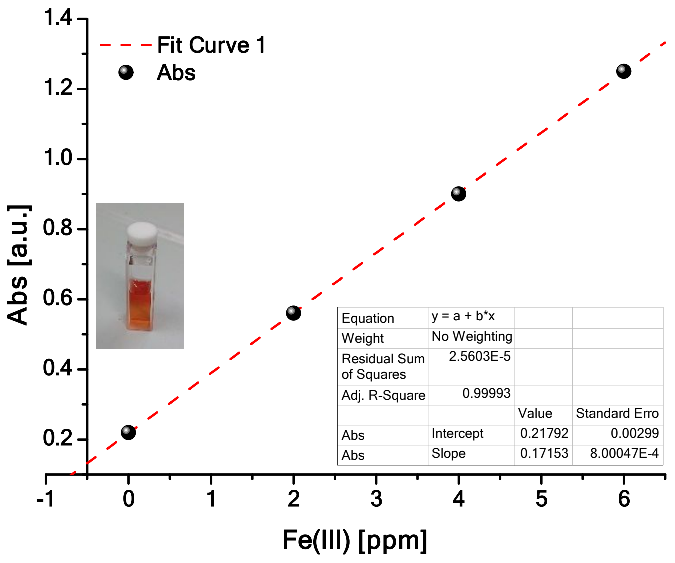

Figure S3. Data manipulation from **Figure S2**.

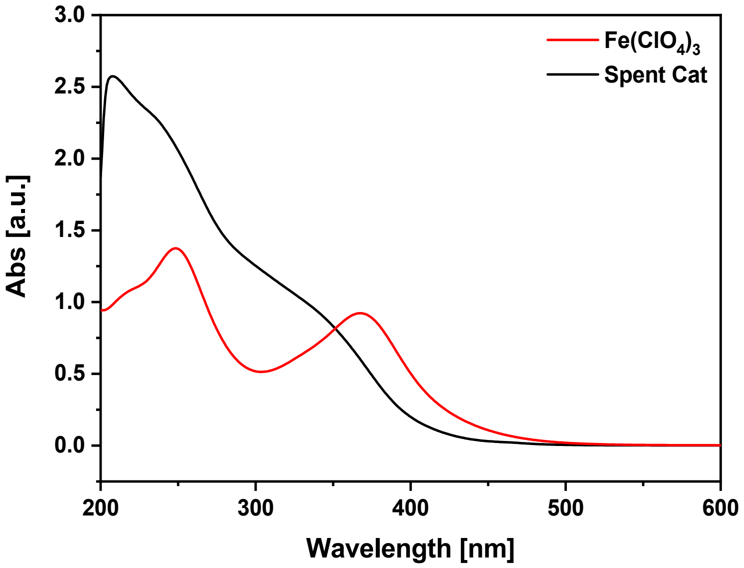

Figure S4. UV-Vis spectra in water and comparison with $Fe(ClO_4)_3$.

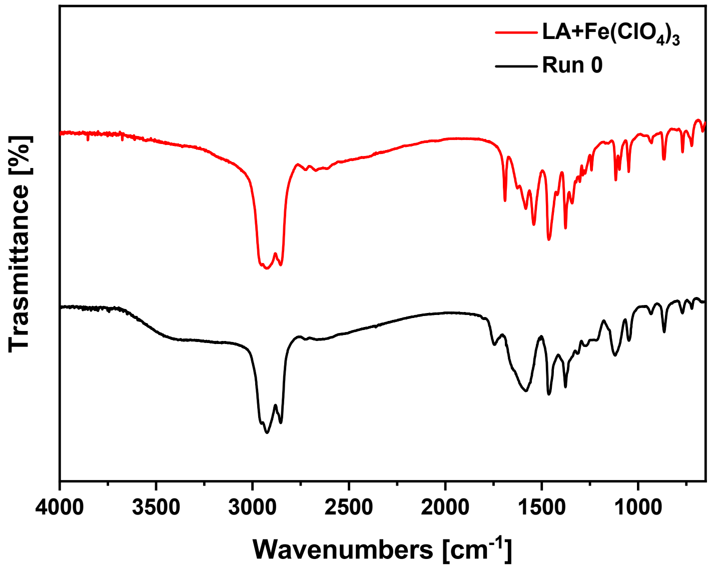

Figure S5. FT-IR spectra and comparison of Run 0 spent cat (0.5 $\%_{mol}$ cat loading) with the resulting solid obtained by treating iron(III) perchlorate with lactic acid (4 h at reflux in petr. eth. bp 40–60 °C).

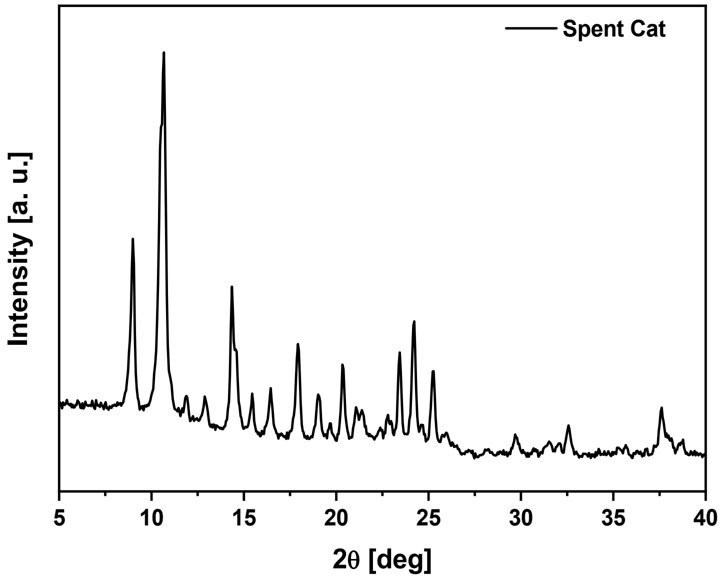

Figure S6. WA-XRD pattern of spent catalyst.

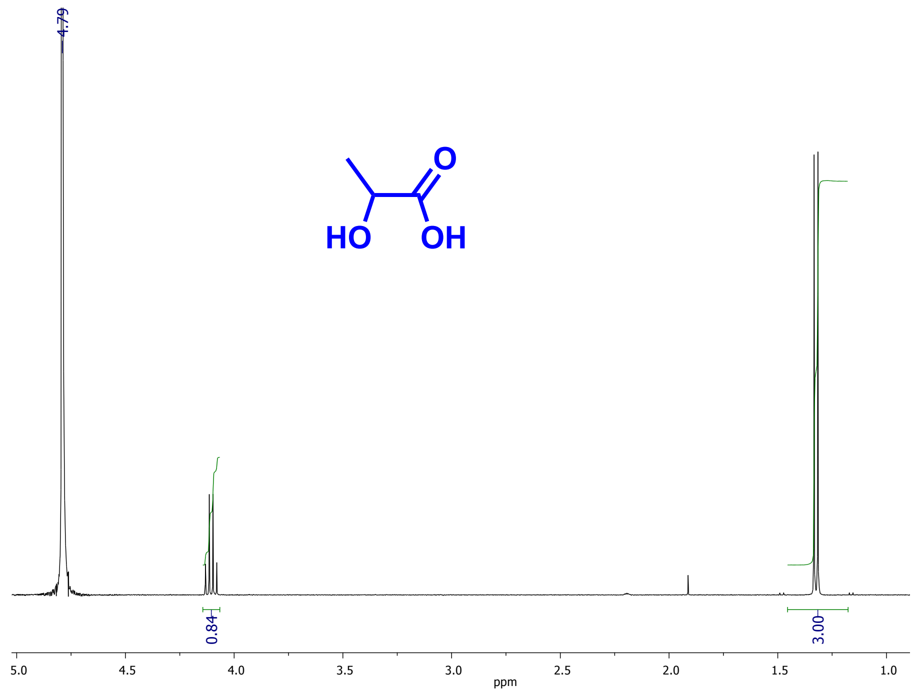

Figure S7. ^{1}H NMR spectra in D_2O of the spent catalyst treated with hydrated $Na_2S{\cdot}9H_2O$.

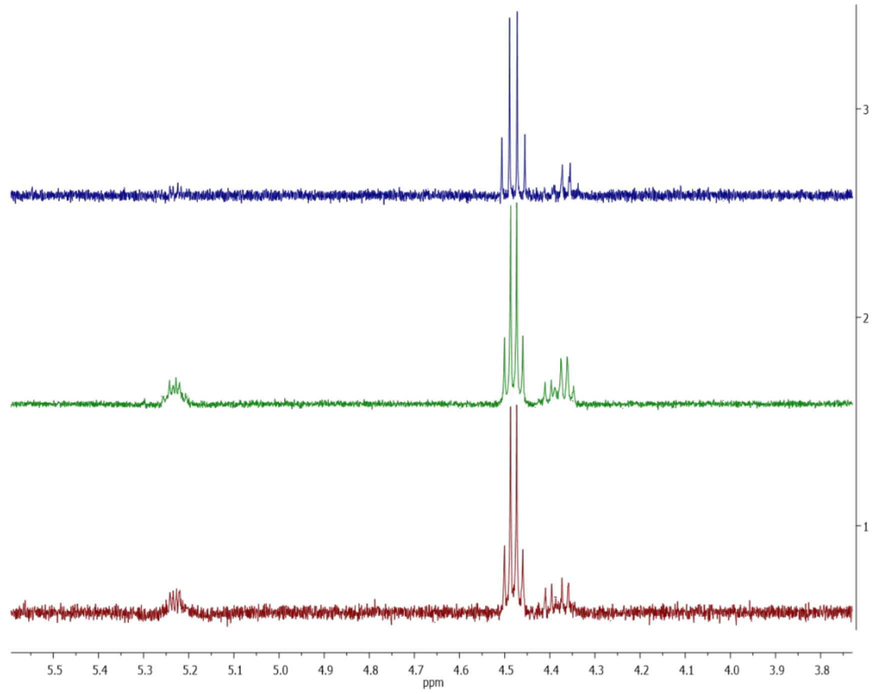

Figure S8. Relevant ^{1}H NMR portion of **Entries 1-3** (bottom to top) from **Table 2**.

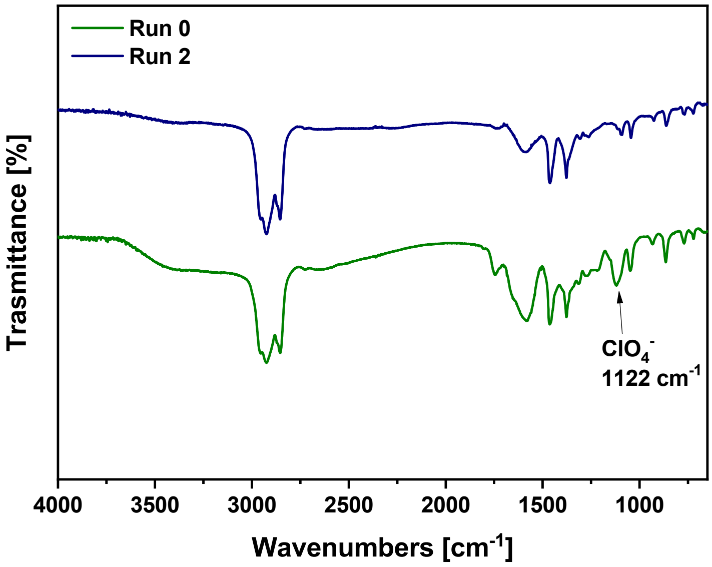

Figure S9. FT-IR analysis after catalytic Run 0 and Run 2 (cat loading 0.5 $\%_{mol}$).

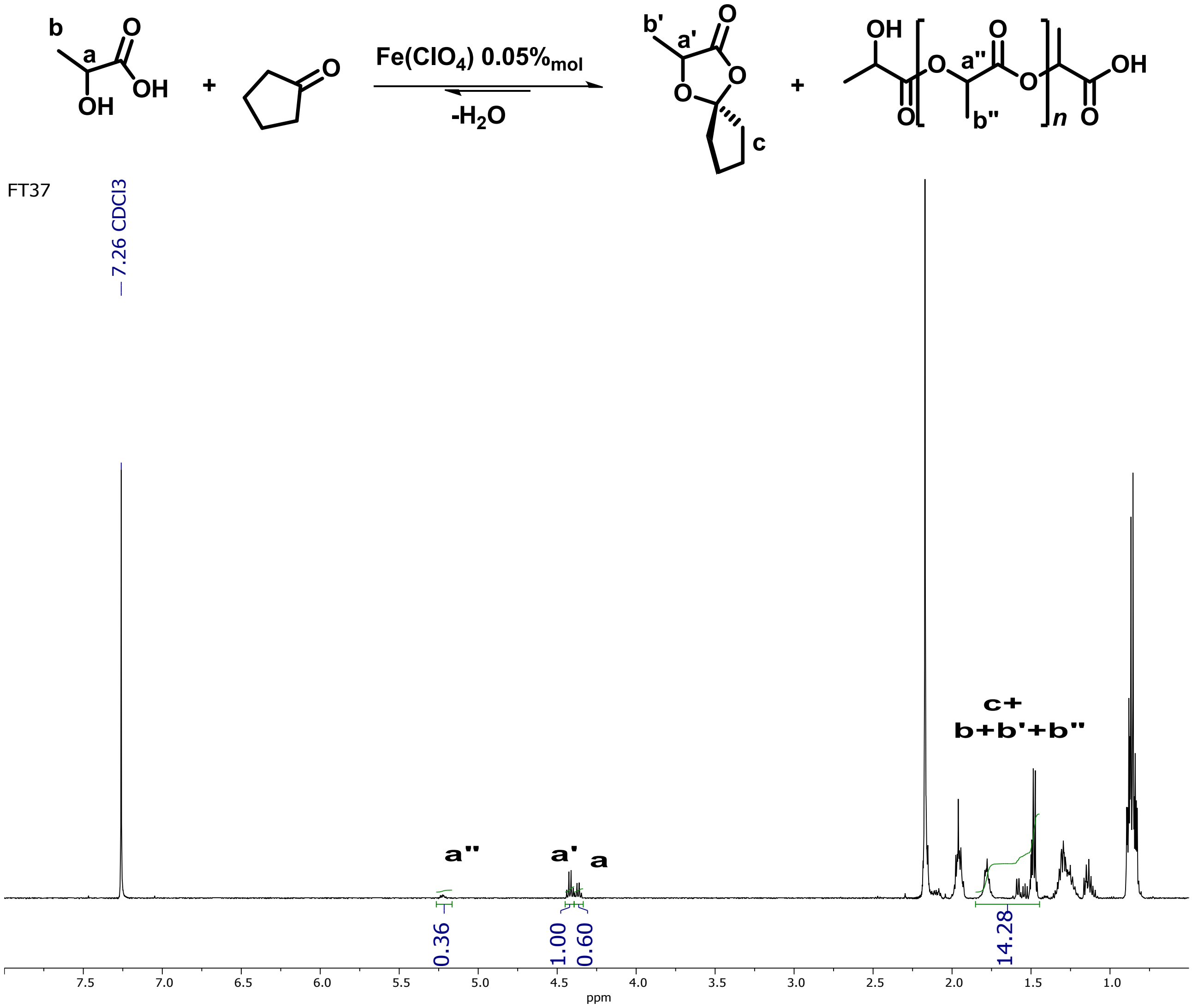

Figure S10. ^{1}H NMR spectrum of **Table 3 Entry 1**, ketalization of lactic acid with cyclopentanone. Reaction conditions: D-S apparatus, reflux 4 h, cat Fe(III) perchlorate hydrate, MR 1, cosolvent petr. eth. bp 40–60 °C. **Used signals**: **a''** 5.20 ppm (m, 1H); **a'**, 4.42 ppm (q, *J* = 6.8 Hz, 1H); **a,** 4.37 ppm (q, *J* = 6.9 Hz, 1H). Multiple signals at 1.8-1.48 ppm have not been attributed to each compound.

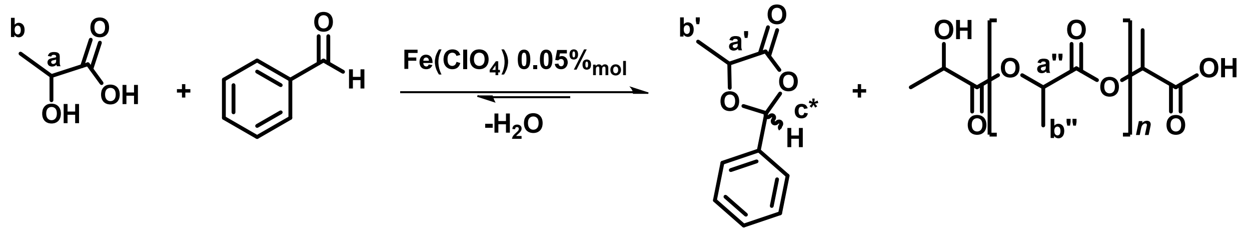

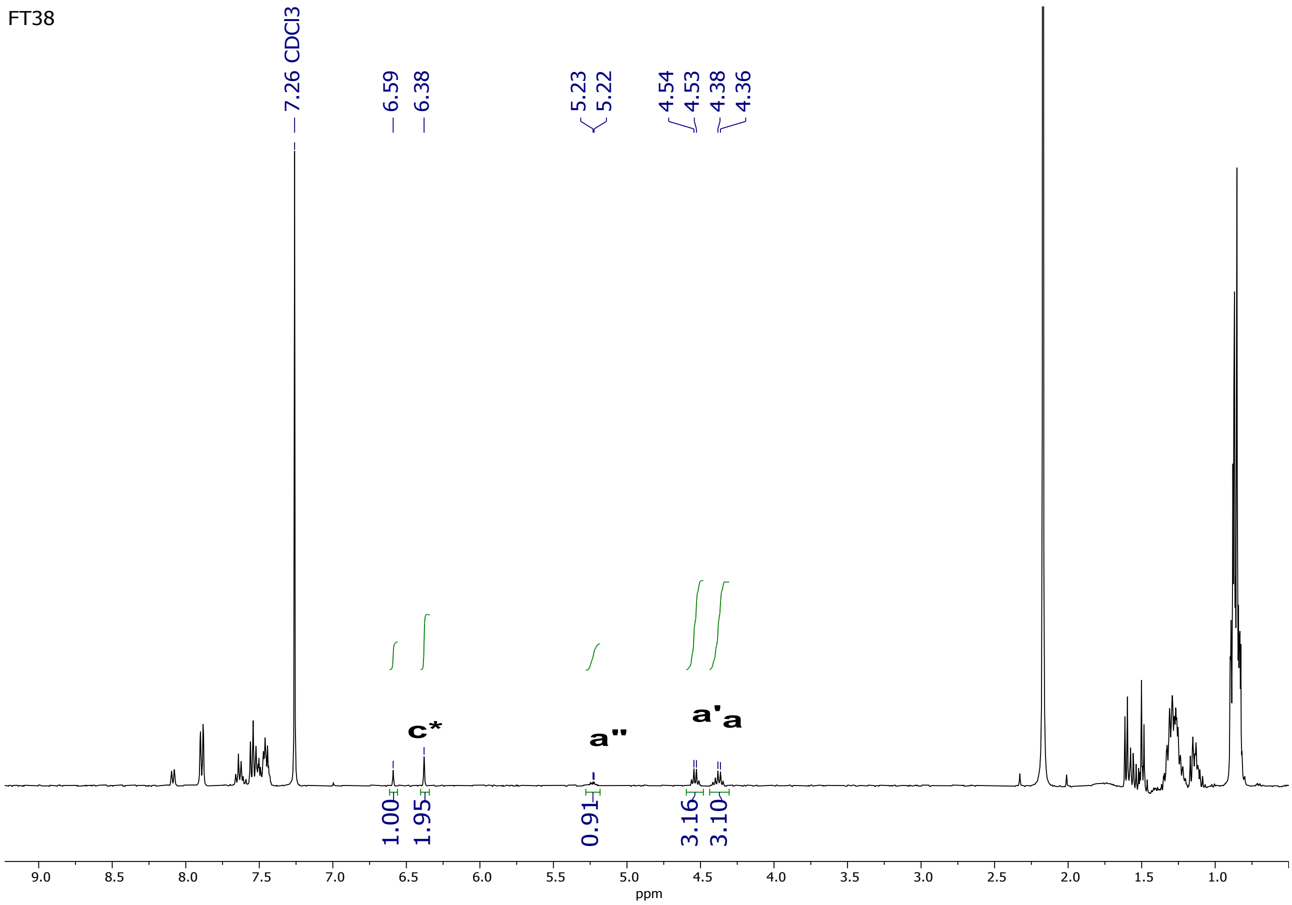

Figure S11. ^{1}H NMR spectrum of **Table 3 Entry 2**, ketalization of lactic acid with benzaldehyde. Reaction conditions: D-S apparatus, reflux 4 h, cat Fe(III) perchlorate hydrate, MR 1, cosolvent petr. eth. bp 40–60 °C. **Used signals**: **c***, 6.59 and 6.38 ppm (s, 1H – minor and major diastereomer); a" 5-23 (m, 1H); **a'**, 4.54 (q, J = 6.6 Hz, 1H); **a**, 4.37 (q, J = 6.9 Hz, 1H).

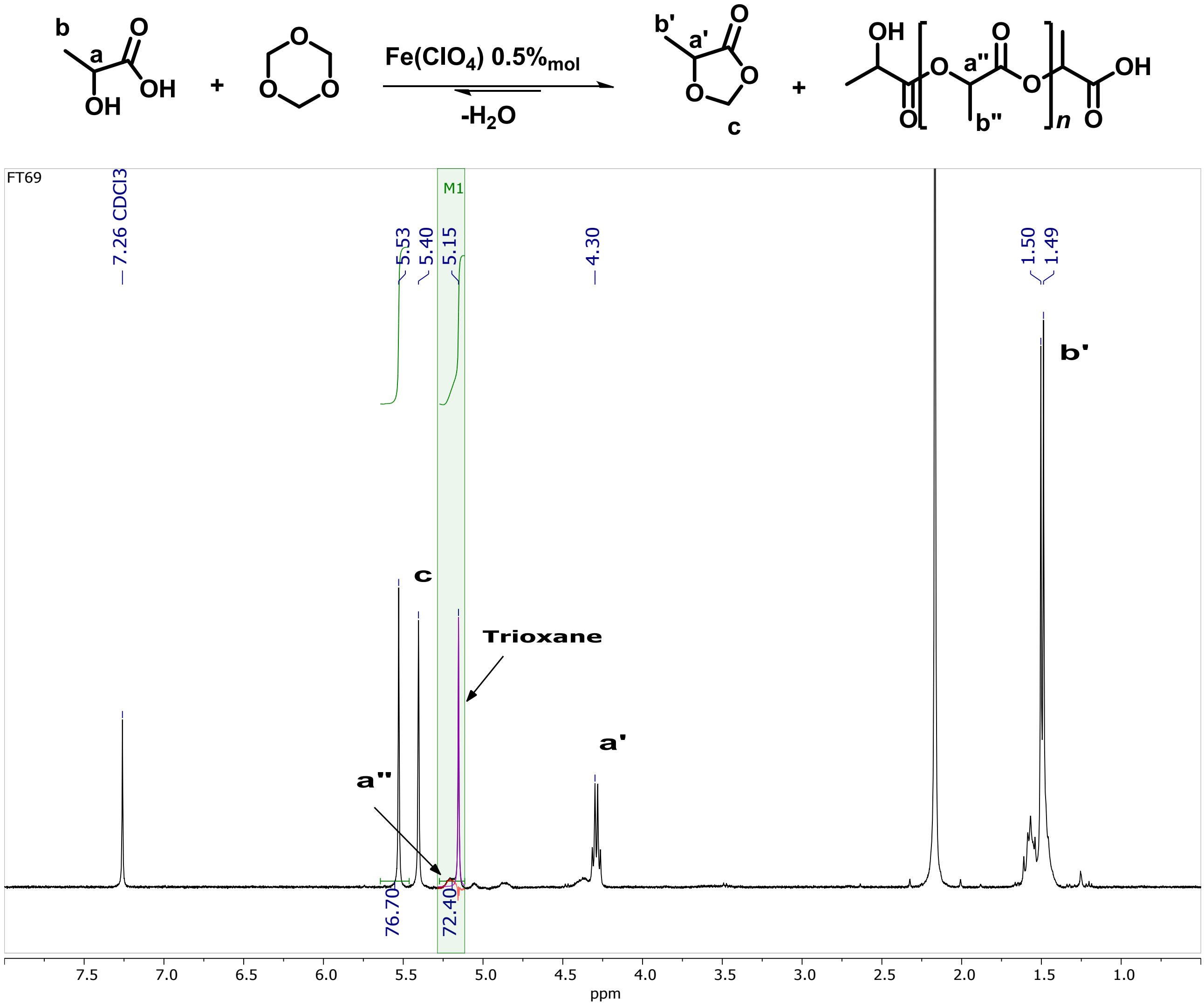

Figure S12. ^{1}H NMR spectrum of **Table 3 Entry 3**, ketalization of lactic acid with trioxane. Reaction conditions: D-S apparatus, reflux 4 h, cat Fe(III) perchlorate hydrate, MR 1, cosolvent petr. eth. bp 40–60 °C. Due to high conversion, volatile compounds were gently evaporated before analysis. **Product signals**: ^{1}H NMR (400 MHz, $CDCl_3$) δ 5.53 (s, 1H), 5.41 (s, 1H), 4.29 (q, J = 6.8 Hz, 1H), 1.50 (d, J = 6.8 Hz, 3H).

Table S2. Fitting data analysis for the ^{1}H NMRspectrum of **Figure S12**.

**Integrals**	**Fitting Area 5.14-5.28 ppm**	**Absolute**	**Normalized**
Total	Trioxane (5.15 ppm) + oligomers (5.20 ppm)	41291.77	72.4
Trioxane	5.15 ppm	32118.44	56.3
Oligomer	5.20 ppm	9173.33	16.1
	Trioxane, %	78	
	Oligomer, %	22	
Product	5.53 ppm		76.7
Lactic acid	4.35-4.40 ppm		n.d.
	Conversion, %		< 99
	Selectivity, %		82
	Yiled, %		81

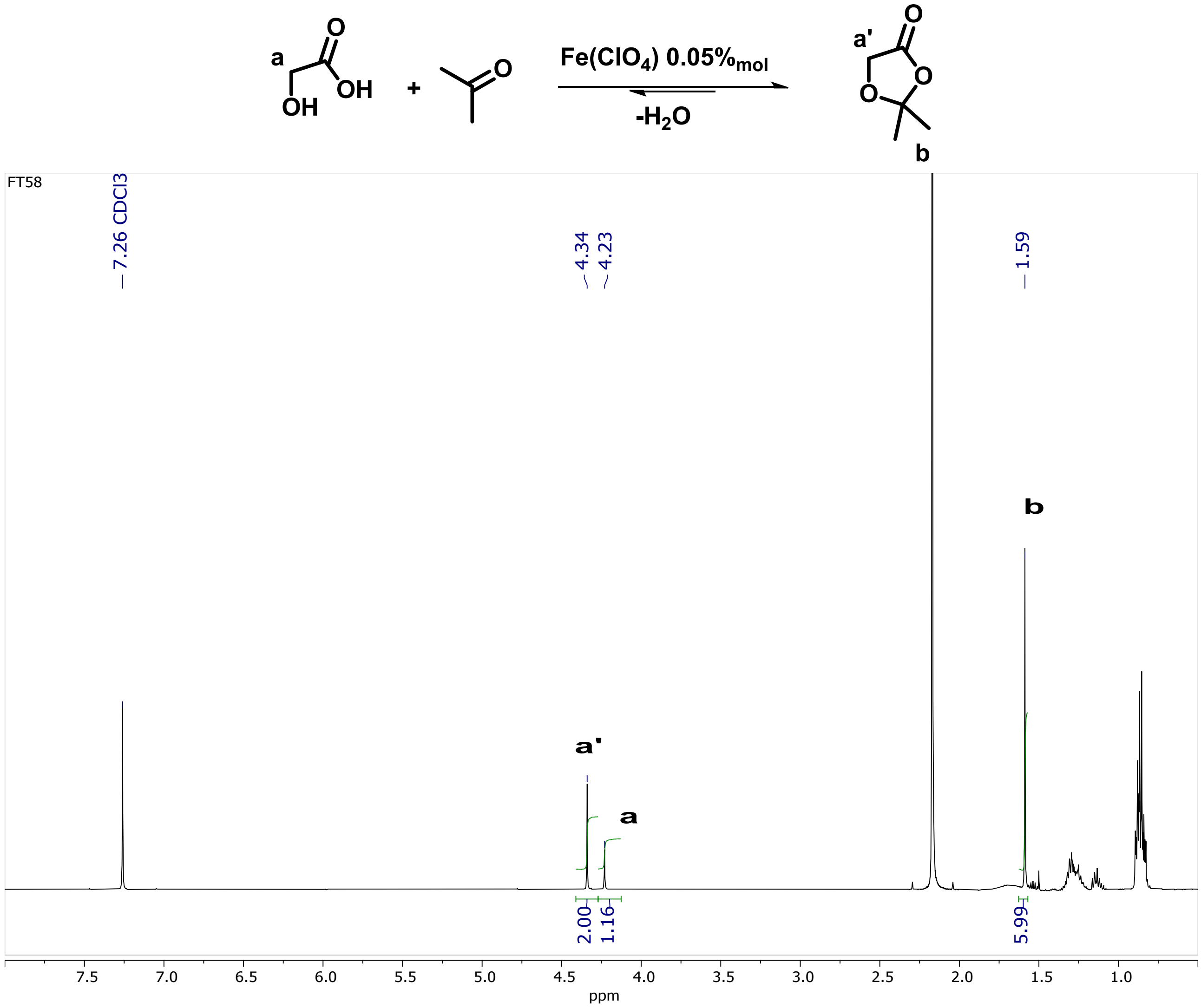

Figure S13. ^{1}H NMR spectrum of **Table 3 Entry 4**, ketalization of glycolic acid with acetone. Reaction conditions: D-S apparatus, reflux 4 h, cat Fe(III) perchlorate hydrate, MR 1, cosolvent petr. eth. bp 40–60 °C. **Product signals**: ^{1}H NMR (400 MHz, $CDCl_3$) δ 4.34 (s, 2H), 1.59 (s, 6H).

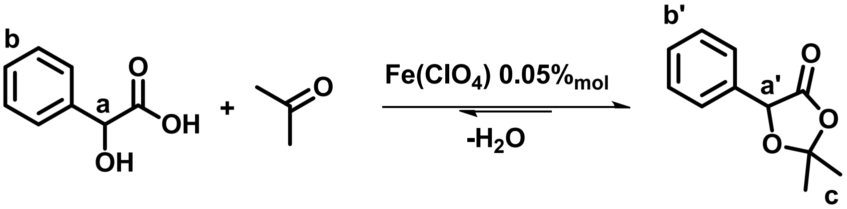

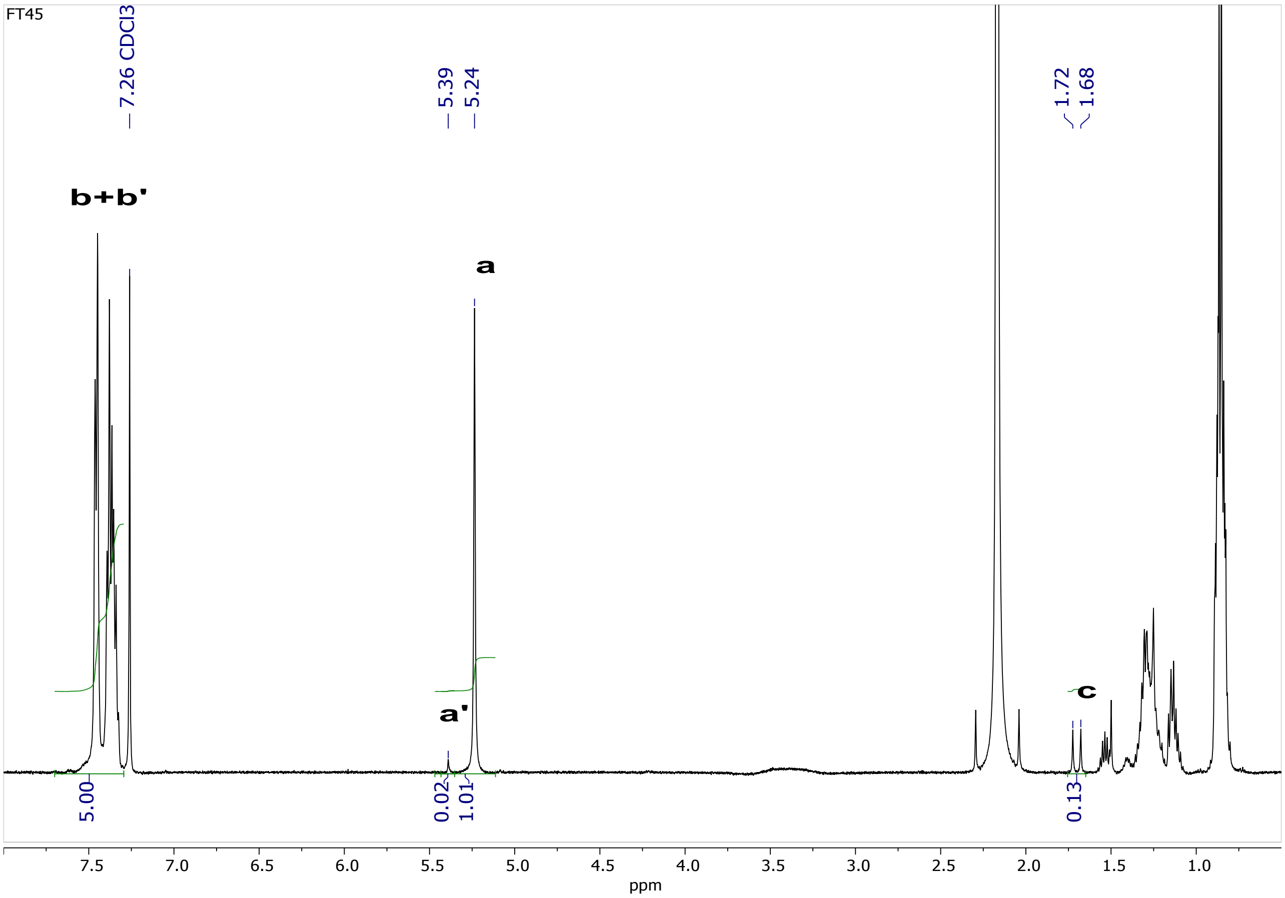

Figure S14. ^{1}H NMR spectrum for the ketalization of mandelic acid with acetone. Reaction conditions: D-S apparatus, reflux 4 h, cat Fe(III) perchlorate hydrate, MR 4, cosolvent petr. eth. bp 40–60 °C. **Product signals**: ^{1}H NMR (400 MHz, $CDCl_3$) δ 7.45 (m, 5H), 5.39 (s, 1H), 1.72 (s, 3H), 1.68 (s, 3H).

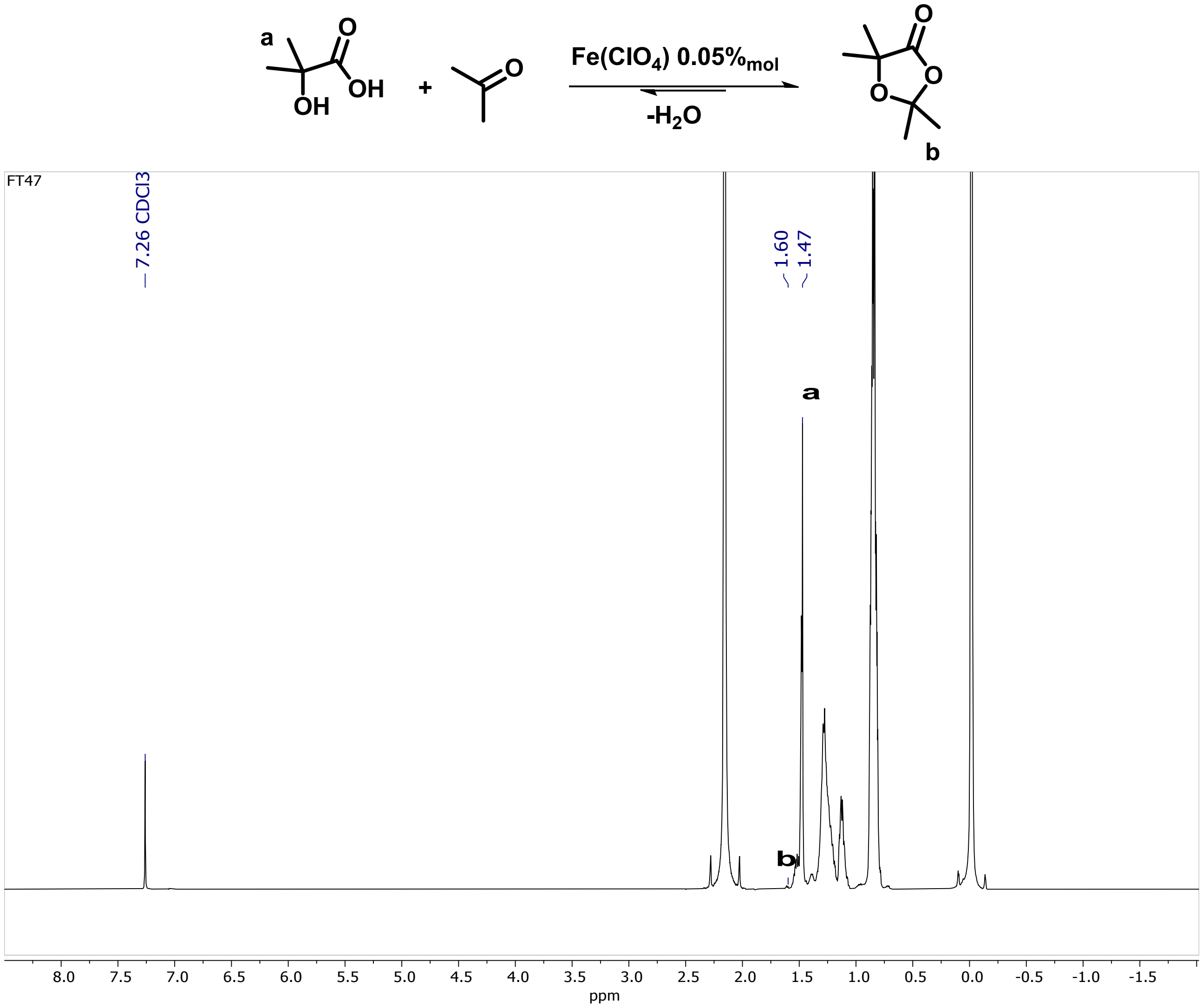

Figure S15. ^{1}H NMR spectrum for the ketalization of α-hydroxyisobutyric acid with acetone.Reaction conditions: D-S apparatus, reflux 4 h, cat Fe(III) perchlorate hydrate, MR 4, cosolvent petr. eth. bp 40–60 °C. **Used signals**: **a** 1.47 (s, 6H – reagent and product); **b,** 1.60 ppm (s, 6H – product).
